# MAGNETIC RESONANCE CHOLANGIOPANCREATOGRAPHY (MRCP) VERSUS ENDOSONOGRAPHY-GUIDED FINE NEEDLE ASPIRATION (EUS-FNA) FOR DIAGNOSIS AND FOLLOW-UP OF PANCREATIC INTRADUCTAL PAPILLARY MUCINOUS NEOPLASMS

**DOI:** 10.1590/0102-672020190001e1471

**Published:** 2019-12-20

**Authors:** Débora Azeredo Pacheco Dias COSTA, João Guilherme GUERRA, Suzan Menasce GOLDMAN, Rafael KEMP, José Sebastião SANTOS, José Celso ARDENGH, Carmen Australia Paredes Marcondes RIBAS, Paulo Afonso Nunes NASSIF, Jurandir Marcondes RIBAS-FILHO

**Affiliations:** 1 Endoscopy Service, Nove de Julho Hospital, São Paulo, SP, Brazil;; 2 AC Camargo Cancer Center, Endoscopy Service, AC Camargo Hospital, São Paulo, SP, Brazil;; 3 Department of Diagnostic Imaging, Paulista School of Medicine, Federal University of São Paulo, São Paulo, SP, Brazil;; 4 Department of Surgery and Anatomy, Hospital das Clínicas, Ribeirão Preto Medical School, Ribeirão Preto, SP, Brazil;; 5 Institute of Medical Research, Evangelical Faculty of Paraná, Curitiba, PR, Brazil.

**Keywords:** Endoscopic ultrasound-guided fine needle aspiration, Pancreatic neoplasms, Diagnóstico, Colangiopancreatografia por ressonância magnética, Aspiração por agulha fina guiada por ultrassom endoscópico

## Abstract

**Background:**

Intraductal papillary mucinous tumor (IPMN) are being diagnosed with increasing frequency. Computerized tomography scanning is commonly used as the primary imaging modality before surgery nonetheless magnetic resonance cholangiopancreatography (MRCP) provides better characterization. Endosonography-guided fine needle aspiration (EUS-FNA) has emerged as a way to reach pathological diagnose.

**Aim:**

To compare results of both methods with surgical pathology findings for classification of IPMN.

**Methods:**

Thirty-six patients submitted to surgical resection with preoperative suspect of IPMN were submitted preoperatively to MRCP and EUS-FNA. Images obtained were analyzed according to a classification determined for each method. ROC curve was used for statistical analysis, that compared the images tests with the purpose of finding the best method for diagnosis and classification of IPMN.

**Results:**

Sixteen patients underwent pancreatoduodenectomy, 16 to subtotal pancreatectomy and only four laparotomy. Pathological diagnosis was IPMN (n=33) and pancreatic intraepithelial neoplasia type 2 (n=3). Twenty-nine revealed non-invasive neoplasia and invasive form in four patients. MRCP and EUS-FNA have correctly diagnosed and classified (type of IPMN), in 62.5% and 83.3% (p=0.811), the affected segment location in 69% and 92% (p=0.638) and identification of nodules and/or vegetation presence in 45% and 90% (p=0.5). Regarding to histopathological diagnosis by EUS-FNA the sensitivity was 83.3%; specificity was 100%; positive predictive value was 100%; negative predictive value was 33.3% and accuracy was 91.7%.

**Conclusions:**

There was no significant difference in the diagnosis of IPMN. However, EUS-FNA showed better absolute results than MRCP to identify nodule and/or vegetation.

## RESUMO

**Racional:**

A neoplasia intraductal mucinosa papilífera (NIMP) está sendo diagnosticada com maior frequência. O método mais utilizado para diagnóstico é a tomografia computadorizada. No entretanto, a colangiopancreatoressonância (CPRM) proporciona melhor caracterização tipo e extensão. A ecoendoscopia com punção por agulha fina (EPAAF), por sua vez, permite o diagnóstico histológico.

**Objetivo:**

Comparar resultados da CPRM e EPAAF com os achados cirúrgicos e patológicos para o diagnóstico e classificação da NIMP.

**Método:**

Foram estudados trinta e seis pacientes submetidos à ressecção cirúrgica por suspeita de NIMP que foram submetidos à CPRM e EPAAF pré-operatórias. Imagens obtidas por ambos os métodos foram analisadas utilizando-se padronização contendo o tipo e a classificação da lesão e os achados foram comparados, tendo como referência a análise patológica do espécime cirúrgico para definir-se qual o melhor método na caracterização do NIMP.

**Resultados:**

Vinte e nove revelaram neoplasia não-invasiva e quatro invasiva. A CPRM e a EPAAF fizeram o diagnóstico e classificaram corretamente (tipo de NIMP) em 62,5% e 83,3% (p=0,811), a localização do segmento pancreático acometido em 69% e 92% (p=0,638) e a identificação da presença de nódulos e/ou vegetações em 45% e 90 % (p=0,5). Quanto ao diagnóstico histológico pela EPAAF a sensibilidade foi 83,3%; especificidade 100%; VPP 100%; VPN 33,3%; e acurácia 91,7%.

**Conclusões:**

Os métodos diagnósticos não apresentaram diferença estatística. No entanto, a EPAAF mostrou resultados absolutos melhores do que a CPRM na identificação de nódulo e/ou vegetação intracístico.

## INTRODUCTION

The increased use of computed tomography (CT), magnetic resonance cholangiopancreatography (MRCP) and endoscopic ultrasonography (EUS) has increased the recognition of pancreatic cystic lesions, classified as neoplastic and pseudocyst^6^ ^,^ ^8^ ^,^ ^21^ ^,^ ^28^. The most common cystic neoplasms are: serous cystic neoplasia, mucinous, solid-cystic pseudopapillary (Frantz) and papilliferous mucinous mucinous intraductal neoplasia (NIMP)^2^ ^,^ ^15^ ^,^ ^31^. The diagnosis and treatment of the latter has evolved since its first description by Ohashi et al.^20^. It is a precursor of pancreatic cancer and has mucin-producing epithelium, which develops preferentially within the main pancreatic duct^29^ and has been increasingly diagnosed^16^ ^,^ ^27^.

Because these lesions vary in type and extent, the ideal examination for adequate characterization needs to be sensitive enough to provide faithful images of extent of damage^6^. In addition, it should provide specific and accurate evaluation in order to establish the differential diagnosis between NIMP and cystic mucinous neoplasia^9^ ^,^ ^10^ ^,^ ^17^.

Despite the development of imaging methods, there is still no ideal for studying this disease. Regarding CT and MRCP, current literature is limited to demonstrate its diagnostic accuracy, as well as to assess the involvement and size of the main pancreatic duct^10^ ^,^ ^23^. CT is used for diagnosis and initial characterization in patients with NIMP^13^ but its use as a single source of images before surgical treatment is common^25^. Waters et al.^33^ believe that CT alone may not be sufficient to establish the diagnosis and accurately determine tumor type and extent, requiring MRCP for complementary analysis. It is observed that the use of other diagnostic methods has been necessary^10^ ^,^ ^19^ ^,^ ^33^. Endoscopic retrograde cholangiopancreatography, endoscopic ultrasonography (mini-probe) and conventional echoendoscopy are invasive methods that can be used with doubtful success for diagnosis^7^ ^,^ ^32^.

Echoendoscopy evaluates the type and extent of NIMP, but according to Waters et al. have blind spots, unable to accurately determine the extent and involvement required for preoperative planning. In addition, it is not widely available method, even in the USA. Contrary to what these authors define, the experience of the present paper has been rewarding. One of the key points is, in addition to allowing the classification and analysis of type and extension, that it provides for the possibility of surgical resection and for obtaining material to characterize the degree of cellular atypia^25^ ^,^ ^33^.

MRCP, unlike the echocardioid puncture, is non-invasive and allows evaluation of the pancreatic duct^33^, providing the same information in the identification of nodules, vegetations, but cannot simultaneously collect material for anatomopathological evaluation^14^.

The objective of this study was to compare the EPAAF with the results obtained by MRCP in the diagnosis and extension of proven NIMP after surgical resection 

## METHODS

This study was carried out at the Department of Endoscopy of the 9 de Julho Hospital, São Paulo, SP, Brazil, and at the Institute of Medical Research of the Evangelical Faculty of Paraná, Curitiba PR, Brazil, approved by the Institutional Ethics Committee under number 53037816.0.0000.0103

### Patients

Patients with suspected NIMP, previously diagnosed by MRCP and/or any other imaging method between January 2010 and September 2015, were referred for diagnostic confirmation by the EPAAF. Of the 298 patients with pancreatic cystic lesions identified on imaging tests, 152 were NIMP.

### Inclusion and exclusion criteria

 Only those who had been diagnosed with NIMP - both during EPAAF and MRCP - and operated with resected material sent for histological analyses, were included.

 Exclusion criteria were those whose final diagnosis was obtained only by EUS, the non-operated who only followed the disease, those who had not previously performed MRCP as part of the protocol for pancreatic disease and those whose MRCP and EUS images were in poor quality and artifacts that affected diagnostic accuracy.

### Patient selection and surgical procedure

Thirty-six patients submitted to surgical resection with preoperative suspicion of NIMP were selected and studied. The data were collected from a prospective database, obtained from the date when the patients had clinical suspicion of NIMP by the imaging tests. All were sent to EPAAF, with the purpose of histological diagnosis and confirmation of NIMP.

The data of both for each patient were recorded focusing: 1) correct diagnosis of the lesion; 2) classification main duct (type I), secondary duct (type II) and mixed (type III); 3) nodules or vegetation; 4) focal disease (a single segment of the pancreas) or multifocal (more than one segment); and 5) place of attachment and extension (head, body and tail).

Twenty-one men and 15 women were enrolled. The mean age at the time of the operation was 62.4 years (11-89). Sixteen were submitted to duodenopancreatectomy, 16 to subtotal pancreatectomy, and four to exploratory laparotomy, because they presented non-resectable tumor (Table 1). The operation was performed in all selected patients, with standardized mean time of three months after puncture.

**TABLE 1:** Demographic aspects and type of surgical treatment imposed on patients with suspected NIMP

Parameters	n
Age, years (range)	62.4 (11-89)
Gender	
Female	21
Male	15
Procedure	
Duodenopancreatectomy	16
Subtotal pancreatectomy	16
Exploratory laparotomy	4

### Equipments

 All EPAAF exams were performed by the same gastroenterologist (JCA) with over 25 years’ experience in diagnostic and therapeutic EUS. We used the Fujifilm ultrasonic platform model SU 7000 and the sectoral echoendoscopic model EG 530UT.

### Parameters evaluated

 Images obtained by both methods were analyzed using a standardized list containing the type and classification of the lesion; anatomical location of the main cyst (head, body and tail); focal and multifocal distribution; existence of communication between cystic lesion and the main pancreatic duct; and identification of nodules and/or vegetation within the cyst or attached to its wall.

Thus, involvement of the main duct was considered when exams showed diameter greater than 0.9 cm in one segment or filling faults inside the duct. All these aspects were considered as additional factors for the diagnostic involvement of the main duct.

### Classification

When the methods identified dilatations of the main pancreatic duct, it was classified as injury of main duct or type I; the involvement of the secondary ducts individually communicating with the main duct of normal appearance (less than 0.5 cm) the neoplasia was classified as originating from the secondary or type II duct. In contrast, the mixed type was classified when there was dilatation of both ducts (main and secondary)^12^ ^,^ ^23^ ^,^ ^30^.

According to the distribution, they were grouped as focal and multifocal, that is, confined to only one surgical site or more than one. From the anatomical point of view, the sites were distributed as: head/uncinate process (right side of the portal vein), pancreas (covering the portal vein), body (left side of the portal vein) and tail (left side to emerging celiac trunk).

### Magnetic resonance cholangiopancreatography

The initial evaluation with MRCP was performed by experienced radiologists in the digestive system and pancreas and the results were compared to the pathological findings of the surgical specimen in all patients with complete data. Only those with good quality were included in this group, without the presence of artifacts that could affect the accuracy of the image diagnosis, following the same standardized classification previously mentioned.

**FIGURE 1:**
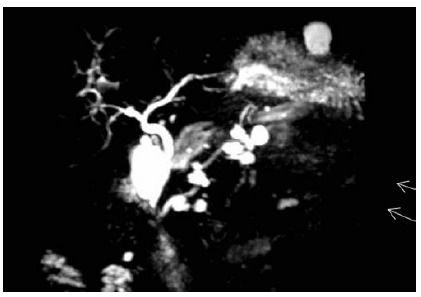
MRCP with contrast (gadolinium): papillary mucinous intraductal neoplasia of the secondary duct (fine white arrows), multifocal located in the head, body and tail.

The MRCPs were performed using high magnetic field equipment (1.5 T) and equipped with body coils. The acquisition sequences were axial weighted in T1 and T2 (with and without fat suppression), the cut thickness being between 5-7 mm. Dynamic axial images were also acquired after paramagnetic contrast (gadolinium, Figure 1).

### Ecoendoscopy associated with fine needle puncture

 EPAAF was performed to evaluate the presence of NIMP, aiming to identify nodules and/or vegetation inside its interior, evaluate the presence of communication between the cyst and the main duct, and perform a puncture to obtain material for anatomopathological study. Patient preparation and control followed the following routine^4^: 8 h fasting, 30 drops of dimethylpolysiloxane, topical anesthesia in the oropharynx with 10% lidocaine spray, peripheral vein puncture maintained throughout the procedure, Propofol 10 mg/ml (Diprivan® - Astrazeneca) intravenously (10 mg/kg) and anticholinergic agents to decrease duodenal motility as needed. Patients always remained in the left lateral decubitus for the examination.

**FIGURE 2:**
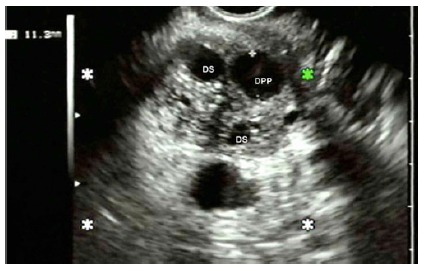
Echoendoscopic image: dilation of the main pancreatic duct (DPP) and secondary duct (DS) in papillary mucinous mucinous intraductal neoplasia type III

The introduction of the device occurred with direct vision until the transposition of the cricopharyngeal muscle and progression blindly to the esophagus, due to the oblique view of the used equipment. The gastric chamber was slightly inflated for progression of the device to the second duodenal portion in the ideal positioning, below the duodenal papilla. Afterwards, the instillation of physiological solution in the duodenum was chosen, enough to improve the image and to diminish the projection of artifacts. Ultrasound images were recorded, and only the best images captured on the video were recorded (Figure 2). The cuts were performed sequentially, withdrawing the device slowly and positioning it at specific points according to the standardization of biliopancreatic pathways, as described by Giovannini et al.^5^. The head of the pancreas, uncinate process, common bile duct, duodenal papilla and gall bladder were visualized and examined through the duodenum. The tail and body of the pancreas, celiac trunk, superior mesenteric artery and adjacent structures were examined through the gastric chamber^28^. The puncture was performed at the end of the procedure after administration of antibiotic prophylaxis with the use of ciprofloxacin hydrochloride 400 mg (Cipro^®^ - Bayer Pharma) intravenously, dose of attack. After this procedure, the patients were under clinical observation for 2 h. After the patient was re-established, he was discharged with a prescription of Cipro^®^ 500 mg orally every 12 h for five days^1^ ^,^ ^5^ ^,^ ^34^.

### Sample classification 

They were classified according to the presence or absence of involvement of the main duct using the criteria of the World Health Organization. The graduation was: adenoma, borderline, high grade or carcinoma “in situ” and invasive. Two patients were classified as grade 2^13^ pancreatic intraepithelial neoplasia (NIPan), even with suspected involvement of the secondary duct.

### Statistical analysis

The results obtained through histological examination were compared with those of the imaging. The agreement between two tests was described as the sensitivity, specificity, positive predictive value, negative and accuracy, considering their estimates and respective confidence intervals estimated by Fisher’s exact test. Sensitivity, specificity, positive/negative predictive value and accuracy of methods for malignant NIMP were established. The ROC curve was used to compare the imaging tests, EPAAF vs. MRCP to find the best diagnostic and classification method for NIMP.

## RESULTS

### Pathological, surgical and classification findings

The pathological examination revealed 33 patients with NIMP. Twenty-nine had a non-invasive type, 16 adenomas, 10 borderline, and three as in situ carcinomas. Four presented the invasive form. The other three were diagnosed as intraepithelial neoplasia (NIPan grade 2, Table 2).

**TABLE 2:** Pathological aspects of NIMP (n=33) and NIPan (n=3)

Parameters	n
Anatomopathological (NIMP)	
Adenoma	16
Borderline	10
Carcinoma in situ	3
Invasive	4
Pathological classification (NIPan)	
Grade 2	3

Twenty-five had involvement in the main pancreatic duct, three of the secondary duct, and eight of the mixed type. Pathological and surgical findings revealed that the lesion was focal in 28 and multifocal in eight. In 21 the results demonstrated the presence of nodules and/or vegetations being adenocarcinoma “in situ” (n=4) and adenoma (n=17). According to the surgical findings and/or pathological lesion, a head injury was demonstrated in 21, in more than one segment in eight and body seven. Of the three patients with type II suspected EUS, all had type 2 NIPan without nodules and/or vegetations (Table 3).

**TABLE 3:** NIMP classification

Classification	Pathology	MRCP	EUS	p
a) Types of NIMP				
Main duct (I)	25	21	25	0.811
Mixed (III)	8	5	6	1
Secondary pipeline (II)	3*	1	3	1
b) Focal or multifocal				
Focal	28	20	27	0.638
Multifocal	8	5	6	1
c) Nodules and vegetation				
Gift	21	10	19	0.5
Absent	15	15	15	1
d) Location of the lesion				
Head	21	17	19	1
More than one segment	8	5	7	1
Body	7	3	7	1

*=Two cases of pancreatic intraepithelial neoplasia

The mean size of the larger cystic lesions was 3.7 cm (0.9-10.5). Twenty-one patients had cystic lesions smaller than 3 cm; five between 3.1 and 5.0 cm and 10 greater than 5 cm.

### Magnetic resonance cholangiopancreatography

**FIGURE 3:**
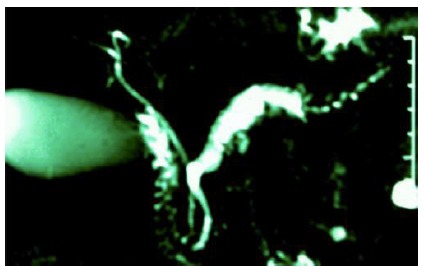
Magnetic resonance cholangiopancreatography: exuberant dilation of the main pancreatic duct identified by white and fine arrows in papilliferous mucinous intraductal type I tumor

 The correct suspicion of NIMP occurred in 27/36 (75%), the identification of mucinous cystadenoma in 6/36 (16.6%) and 3/36 (8.3%) was normal. The correct type I diagnosis was done in 21/25 (84%, Figure 3), type II in 33.3% and type III in 5/8 (62.5%). It showed accurately that the lesion occupied only one anatomical site of the pancreas (focal) in 20/28 (71.4%) and more than one site (multifocal) in 05/08 (62.5%). It revealed nodules or vegetation in 10/21 (47.6%). He identified the exact location of the tumor in 30/36 (83.3%). In 2/4 (50%) patients, it was able to identify the invasive form of this neoplasia. The sensitivity, specificity, positive predictive value, negative and accuracy of this diagnostic method, considering their estimates and respective confidence intervals (CI) of 95%, were 62.5% (40.6% - 81.2%), 90.5% (77.9-100%), 88.2% (63.6-98.5%), 86.4% (72-100%) and 80.8% (65.6-95, 9%).

### Ecoendoscopy associated with fine needle puncture

 The diagnostic suspicion was NIMP in 27/36 (75.4%), mucinous cystadenoma in 5/36 (13.8%), serous cystadenoma 3/36 (8.1%), cystadenocarcinoma in 1/36 (2, 7%). The correct diagnosis of type I, II and III, occurred in 100%, 100% and 75%, respectively and correctly showed a focal lesion in 27/28 (96.4%) and multifocal lesion in 6/8 (75%).

**FIGURE 4:**
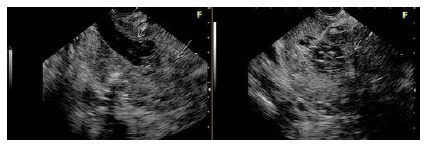
A) Echoendoscopic image of the dilatation (0.85 cm) of the main pancreatic duct (short white arrow) in the head of the pancreas and distal portion and vegetation inside (long white arrow); B) 22G needle puncture time to collect material from the interior of the main pancreatic duct and vegetation (short white arrow).

The vegetations or nodules were identified within the cyst or adhered to its wall in 19/21 (90.9%) patients (Figure 4). It correctly identified the exact location of the lesion in 33/36 (91.6%) and the invasion in 4/4 (100%).

The sensitivity, specificity, positive predictive value, negative and precision of puncture echoendoscopy, considering their estimates and their respective 95% CI were 80% (44.9-100%), 95.2% (86.1% 100%), 80% (44.9-100%), 95.2% (86.1-100%), 92.3% (82.1-100%), respectively.

### Comparison between EPAAF and MRCP for diagnosis and classification of NIMP

**FIGURE 5:**
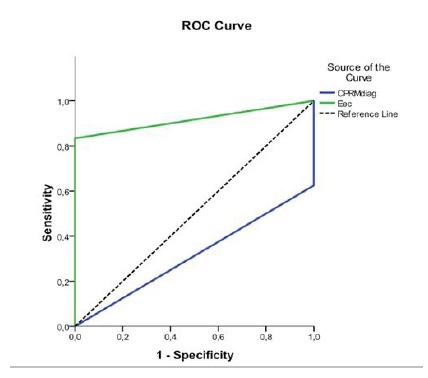
ROC curve comparing EUS and MRCP for NIMP diagnosis and evaluation

 The ROC curve demonstrated that EUS revealed better accuracy when compared to MRCP, but the test were invalidity: its results were no better than chance. EUS presented greater sensitivity and specificity when compared to MRCP in the identification and classification of nodules and/or vegetations within the cystic areas of NIMPs (Figure 5).

## DISCUSSION

The diagnosis and treatment of NIMP remains controversial^21^ ^,^ ^33^. With the quality and availability of current imaging studies, in addition to a better understanding of physicians, the disease has been increasingly diagnosed^24^ ^,^ ^26^ ^,^ ^27^. Thus, it is important for the surgeon to apply the best possible diagnostic method through imaging tests available in clinical practice to accurately determine the diagnosis, characterization, extent, and type of tumor^2^. These factors have important implications, especially in the elderly with comorbidities, to establish a correct stratification of the risk of developing pancreatic cancer and to determine the best treatment in an individualized way^16^ ^,^ ^28^.

This study was performed in patients who had a strong suspicion of NIMP by MRCP. All patients were referred for echocardiographic puncture and then confirmed the results by obtaining a sample for pathological examination. Each image was carefully studied and the main information was recorded by medical specialists in radiology and gastroenterology, to compare the results of the operation with pathological findings. The entire study followed standardized criteria, because this disease can evolve over time and change its type and size. Therefore, the patients underwent surgical treatment within three months after the analysis by these imaging exams. According to this study, both are excellent methods for the diagnosis and classification of this neoplasm with similar results. However, echocardiographic puncture has advantages over MRCP, which are: 1) precise diagnosis of the degree of cellular atypia; 2) precise determination of nodules and/or vegetation; 3) identification of the extent of the disease. These data corroborate with Martin’s study^18^.

The diagnosis of NIMP can be clinical or pathological. The clinic is based on history, endoscopic findings, cytology obtained by endoscopy, and radiological findings^2^. From the radiological point of view, the diagnosis is made through the identification of the dilatation of the main pancreatic duct described. The correct classification of the pathological type has major implications for the stratification of patients who are at risk of developing pancreatic cancer. Knowledge of this fact also determines the best treatment for them^14^.

The malignant potential involving NIMP of the main duct is larger in relation to the mixed type and practically non-existent when it affects the secondary duct^22^. Thus, involvement of only the secondary duct provides a lower risk of pancreatic cancer. Furthermore, in the present study, three cases identified as type II NIMP in the imaging were not confirmed as such, but as grade 2 pancreatic intraepithelial neoplasia, accompanied by dilation of secondary ducts. This finding is similar to that described by other authors^33^. It is noteworthy that in this study the puncture suspected NIPan in two cases, while the images obtained by MRCP and the EUS of type II NIMP with small dilation of the secondary duct.

Dilation of the main duct as an isolated tumor component may be the most important radiographic criterion of high risk for invasive cancer or risk of malignancy^33^. Thus, in this study, both were effective in classifying the type correctly. This is especially true in patients with comorbidities and low-risk lesions who could have their tumor controlled through periodic follow-up instead of receiving surgical treatment.

The extent of the disease also has significant implications, both for the stratification of cancer risk and for making the right decision on surgical resection margins^15^, in order to prevent relapse^3^ ^,^ ^35^. In addition, new evidence suggests that mixed-type multifocality is associated with malignancy. Although MRCP is sensitive to detect small lesions on secondary ducts, it presented inferior results when compared to the EPAAF to identify lesions smaller than 0.5 cm.

The rate of relapse after surgical resection may be influenced by the sensitivity in detecting the extent of the disease in the preoperative period^11^ ^,^ ^36^. In this study, there were only two recurrences after the Whipple procedure identified by MRCP and confirmed by the EPAAF, requiring total pancreatectomy of the remaining gland. Another point to be discussed is that this may give a false idea of a high rate of recurrence due to a miscalculation in the extent of preoperative disease, when in fact the lesions were too small to be recognized in imaging tests. This aspect opens the door to new clinical research where the use would be part of the research by absorbing patients with suspected disease, as it can, with high accuracy, identify tiny cystic areas not seen by other methods.

The guidelines of this consensus on mucinous cystic neoplasia and NIMP state that MRCP is the best method to describe the appearance of lesions and is useful for determining communication with the ductal system^5^ ^,^ ^34^. This study demonstrates that, in addition to EUS to determine results similar to other methods to evaluate these parameters, it also more accurately identifies the presence of nodules and vegetations (predictors of malignancy) and presents a sensitivity rate of 80% for histological diagnosis. This international consensus suggests that mucinous cystadenoma should be resected and asymptomatic patients with type II NIMP smaller than 3 cm can be observed safely. In addition, it also determines that MRCP is equivalent to CT for investigation of pancreas looking for small secondary ducts

Based on this careful selection of patients with the disease and data analysis, it seems clear that MRCP has a high resolution for the planning and adoption of therapeutic measures^14^, but in addition, the preoperative propaedeutics by EPAAF becomes useful, due to the high sensitivity to detect nodules/vegetations, besides confirming the diagnosis of malignancy (sensitivity of 80% of the puncture)^25^. Previous studies have not adequately addressed this issue, targeting the best modality for NIMP management, and no published study comparing these diagnostic methods was found. They are found in the literature evaluating several isolated imaging methods^8^ ^,^ ^12^. However, Kawamoto et al.^12^ justifies that there is no indication for puncture due to the existence of blind spots in the echoendoscopy, in addition to not assessing the extent of the disease satisfactorily. However, the opposite was demonstrated, being effective to identify the type (I, II and III), multifocality and, even better if compared to MRCP to show nodules or vegetations, in absolute numbers, despite not presenting statistical difference (p=0.5). Thus, it is believed that this must be done before performing a surgical procedure^10^.

Sahani et al.^23^ demonstrated CT and MRCP accuracy to assess the involvement of the main duct. They demonstrated their sensitivity to identify the communication between the cystic lesion and the main pancreatic duct. The sensitivity found was 83% and 87%, respectively. In addition, the diagnostic performance of CT and MRCP to determine the malignant potential of NIMP was similar and agreed, suggesting that follow-up with both modalities can be used^10^.

The international consensus guidelines for pancreatic cystic lesions report that the diameter of the main duct greater than 1 cm strongly suggests this disease^30^. Signs of chronic pancreatitis occurred in many patients, evidenced by tortuosity and dilation of the main duct. In addition, the presence of mucin plugs downstream may result in obstruction and dilatation thereof upstream. Any of these factors can lead to misclassification and overestimate the diagnosis. To avoid this problem, the authors indicated the echo-guided puncture to confirm the diagnosis by obtaining fragments of dilated main duct^1^. There is no doubt about the accuracy of these diagnostic modalities to determine the diameter, but the images provided even for one segment, the latter through the eco-guided puncture, allow better characterization as evidenced by its similarity with findings from pathological studies. In the data of this series related to ductal communication, they were based on the radiological and ultrasonographic analysis performed before the histological diagnosis. Thus, it can be observed that the results of both were similar.

EUS is considered the gold standard examination for pancreatic investigation, providing data on the morphology of these lesions and enabling, through real-time fine needle guided puncture, the collection of material for histological evaluation and tumor biochemical markers. It is known that NIMP has malignant potential and malignancy indications are: pancreatic duct involvement, dilatation above 5 mm, cystic cavity greater than 30 mm, presence of murine nodules, existence of a tissue component developed at from a cystic lesion and the presence of lymph nodes. Complete resection is recommended, especially for the main duct NIMP, and especially in the presence of symptoms. The risk of malignancy of secondary ductal NIMP is lower, suggesting that surveillance may be sufficient to avoid functional loss of the pancreas associated with surgical resection^33^. Increasing efforts have been made to identify predictors of malignancy and avoid indications of unnecessary secondary ductal NIMP surgical resection, resulting in a second set of recommendations from the international consensus guidelines, the Fukuoka International Consensus, published in 2012.

The guidelines of this consensus on mucinous cystic neoplasia and NIMP state that MRCP is the best method to describe the appearance of lesions and is useful for determining communication with the ductal system^5^ ^,^ ^34^. This study demonstrates that, in addition to EUS to determine results similar to other methods to evaluate these parameters, it also more accurately identifies the presence of nodules and vegetations (predictors of malignancy) and presents a sensitivity rate of 80% for histological diagnosis. This international consensus suggests that mucinous cystadenoma should be resected and asymptomatic patients with type II NIMP smaller than 3 cm can be observed safely. In addition, it also determines that MRCP is equivalent to CT for investigation of pancreas looking for small secondary ducts of small proportions^29^. These guidelines, however, do not directly address the best imaging modality to be used preoperatively for accuracy of diagnosis, type and extent of disease. In addition, it cannot be forgotten that three patients with type II suspicion (with lesions smaller than 3.0 cm) were diagnosed with NIPan and according to the guidelines these patients should be followed by imaging.

Thus, in view of the importance of a correct follow-up for NIMP, its validation may generate new studies^31^, encouraging the greater applicability of the EPAAF for the diagnosis and follow-up of these pancreatic cystic neoplasms.

## CONCLUSION

Both studied methods did not have significant statistical difference for the diagnosis and determination of the extent of NIMP. However, the EPAAF revealed better absolute results to identify nodules and/or vegetations, besides providing histological diagnosis and being essential for the management of the disease.
